# The Impact of Physiological and Psychological Fatigue on Work Efficiency: A Case Study of Parcel Sorting Work

**DOI:** 10.3390/s24185989

**Published:** 2024-09-15

**Authors:** Miaomiao Li, Zuqin Ma, Rui Yan, Jielin Yin

**Affiliations:** 1 School of Economics and Management, Beijing Information Science and Technology University, Beijing 100192, China; miaomiaoli916@bistu.edu.cn (M.L.); yinjielin0924@bistu.edu.cn (J.Y.); 2 School of Economics and Management, University of Science and Technology Beijing, Beijing 100083, China; yanrui@ustb.edu.cn

**Keywords:** parcel sorting, physiological fatigue, psychological fatigue, work efficiency, electromyography (EMG), electrodermal activity (EDA), intermittent rest, labor quota

## Abstract

The popularity of online shopping in China has increased significantly, creating new development opportunities for the express delivery industry. However, the rapid expansion of the express industry has also created challenges in the parcel sorting process. The demanding nature of parcel sorting work, which is characterized by intense and prolonged repetitive tasks, makes individuals particularly vulnerable to the effects of fatigue. Fatigue is a complex condition that encompasses both physiological and psychological exhaustion. It often results in reduced energy levels and diminished functionality, significantly impacting an individual’s performance at work and their overall well-being. This study aimed to investigate how physiological and psychological fatigue affects sorting efficiency and to identify appropriate rest periods that will allow employees to maintain their performance levels. The research involved fifteen participants who took part in a 60 min continuous sorting experiment and a similar experiment with scheduled breaks. During both trials, we collected data on participants’ electromyography (EMG) and electrodermal activity (EDA), as well as subjective fatigue ratings (RPE). Signal features such as the median frequency (*MF*) of EMG and the skin conductance level (*SCL*) were analyzed to assess physiological and psychological fatigue, respectively. The results show that physiological fatigue mainly affects sorting efficiency in the first 30 min, while psychological fatigue becomes more influential in the following half-hour period. In addition, subjective fatigue levels during the first 30 min are primarily determined by psychological factors, while beyond that point, both physiological and psychological fatigue contribute to subjective fatigue. Rest periods of 415–460 s, based on EDA recovery times, effectively support sorting efficiency and participants’ recovery. This study highlights the complex ways in which fatigue affects parcel sorting performance and provides valuable theoretical and practical insights for establishing labor quotas and optimizing work schedules in the parcel sorting industry.

## 1. Introduction

The express industry plays a pivotal role in China’s service sector, functioning as a crucial conduit between the supplier and the consumer. It is a principal industry that has transformed distribution methods and facilitated upgrades in consumption patterns. The growth of internet shopping has significantly boosted online consumption, thereby creating new opportunities for the parcel industry, which is closely intertwined with e-commerce [1]. In the express industry, parcel sorting employees are typically required to work for periods longer than 8–12 h per day. There have been reports in the news of tragic incidents involving employees that have suffered sudden death due to overwork. This can be attributed to long working hours and excessive fatigue. Fatigue is a complex physiological and psychological state that results in physiological and psychological energy depletion and functional decline. This state has a significant negative impact on individuals’ work efficiency and overall health [2,3], especially in the case of end-stage parcel sorting work, where fatigue-related issues are particularly prominent. The rapid growth of the express delivery industry in China has led to a substantial increase in the total number of parcels delivered annually [4]. As part of logistics, express sorting affects the timeliness of package delivery and people’s online shopping experience. Additionally, express terminal sorters are often overworked, and their rest periods need to be scientifically determined.

The parcel sorting workers are confronted with the challenging task of performing repetitive, high-intensity work for extended periods on a daily basis, which can result in severe physiological and psychological fatigue [3,5,6]. The term “physiological fatigue” is used to describe the depletion of energy, muscle fatigue, and a decline in functionality caused by prolonged physiological activity or work [7]. Wim Ament and Gijsbertus J. Verkerke demonstrated that physiological fatigue can result in the accumulation of metabolic by-products in muscle activity, depletion of muscle glycogen, alterations in muscle membrane structure, reduced efficiency in neuromuscular transmission, and modifications in the central nervous system, including increased brain temperature and alterations in the serotonin system. These changes impact endurance and performance [8]. Moreover, prolonged physiological fatigue markedly elevates the likelihood of developing a range of chronic illnesses and exerts a detrimental influence on the overall health and well-being of workers [9]. Studies indicate a high prevalence of work-related musculoskeletal disorders (WMSDs) among workers. For example, Jingjing Wang et al. found that the prevalence of WMSDs among Chinese airline baggage handlers was particularly high, with lower back pain affecting 62.7% of workers [10]. Personal habits and postural factors were found to be associated with this discomfort. Similarly, research by Corbeil et al. found that obese workers had significantly higher peak torque values at the L5/S1 joint compared to workers of a healthy weight, suggesting greater strain on the musculoskeletal system during manual material handling tasks [11]. In the context of parcel sorting work, the constant handling and sorting of parcels places significant stress on the muscular and skeletal systems of the workers [12,13,14]. Previous studies on work fatigue have primarily focused on the detection of physiological fatigue, typically measuring muscle activity and cardiac load using electromyography (EMG) and electrocardiography (ECG) [15,16,17].

On the other hand, psychological fatigue occurs as a result of prolonged mental tension and stress, leading to a decline in cognitive function and emotional changes [18]. Neglecting to monitor the emotional well-being of employees in the workplace can have detrimental effects on several aspects of organizational performance [19]. This oversight can result in decreased work efficiency, increased mental health issues, decreased job satisfaction, impaired decision-making, escalation of conflicts among colleagues, increased healthcare costs, and stifled innovation and creativity within the organization. The physiological demands of parcel sorting are considerable, requiring not only strength but also the capacity for sustained attention and rapid decision-making. This high-intensity mental load can result in many issues, including decreased concentration, slow reactions, and decision-making errors, all of which are indicative of psychological fatigue [20,21,22,23]. The majority of studies examining changes in work-related psychological fatigue have employed rating scales, including The Chalder fatigue scale (CFQ 11) [24], the Karolinska Sleepiness Scale (KSS) [25], Positive and Negative Affect Schedule (PANAS) [26], and the Fatigue Severity Scale (FSS) [27]. However, these methods are susceptible to high subjectivity, significant individual variations, and low accuracy, thereby presenting challenges in real-time fatigue detection [3].

Furthermore, periods of rest play a key role in alleviating fatigue and restoring optimal work efficiency [28]. Short rest periods can assist workers in regaining physiological strength, reducing muscle fatigue, and enhancing attention and decision-making abilities by regulating psychological states [29]. For example, Francesco Fischetti et al. conducted an experiment investigating the effects of a 10 min rest period on the attention and executive functions of healthcare workers. The findings indicated that brief periods of activity effectively enhanced cognitive performance during work [30]. Maarten A.S. Boksem et al. conducted an experiment that investigated the impact of psychological fatigue on behavioural performance, specifically focusing on the role of action monitoring in maintaining behavioural coherence and adaptability [31]. Their findings suggest that enhancing motivation levels may help to mitigate the effects of fatigue, reinforcing the significance of understanding and managing psychological fatigue in work environments. However, there is a lack of research examining the effects of short rest periods on work efficiency and physiological fatigue status in parcel sorting work, particularly studies that holistically consider the impacts of both physiological and psychological fatigue, as well as research on the optimal duration of rest periods.

Applying EMG and electrodermal activity (EDA) to assess physiological and psychological fatigue can facilitate a more comprehensive understanding of fatigue. EMG enables precise measurement of muscle activity and fatigue, whereas EDA reflects psychological stress and emotional changes [32,33,34,35,36]. These objective physiological and psychological measurement methods will facilitate a more comprehensive understanding of fatigue in parcel sorting work and exploration of the efficacy of rest in alleviating fatigue.

This study’s principal objective is to quantify physiological and psychological fatigue changes during parcel sorting tasks by utilising EMG and EDA. Additionally, we aim to investigate the impact of short rest periods on work efficiency and physiological fatigue status. Conducting research in China provides access to relevant data and participants, facilitating a deeper analysis of fatigue’s influence on work efficiency. Through this research, our objective is to provide scientific evidence to enhance the working environment and health conditions of parcel sorting workers, as well as to offer valuable insights for formulating rational rest schedules.

## 2. Materials and Methods

### 2.1. Participants

To create a realistic simulation of a parcel sorting environment, this study was designed to reflect the actual operational conditions observed in express delivery terminals. The gender ratio of sorting staff is typically 4:1.Consequently, participants were recruited through online platforms and subsequently selected through phone screenings, resulting in a final cohort of 12 male and 3 female participants, which reflected the authentic gender ratio of the staff. All participants were enrolled in college and had previously engaged in parcel sorting activities.

To guarantee the overall health of the participants and to eliminate any potential confounding factors, we established exclusion criteria. These criteria included self-reported neurological or medical conditions, thereby ensuring that all participants were in good health and free from cervical spondylosis, muscle soreness, and other related symptoms. Furthermore, participants were required to have no recent history of sports injuries within the past six months, refrain from engaging in vigorous exercise in the two days preceding the experiment, possess a body mass index (BMI) within the normal range, and obtain a minimum of seven hours of sleep before the experiment, without the use of medication, and to abstain from alcohol consumption for one month prior to the experiment.

### 2.2. Experiment Procedure

To replicate the work tasks conducted in a parcel sorting center, participants were engaged in two different work modes during the experiment. In Experiment A, participants were engaged in continuous sorting tasks without any intermissions, whereas in Experiment B, they were involved in intermittent work. In Experiment B, participants engaged in sorting activities for 30 min, after which they were permitted a rest period until their physiological signals returned to a stable state, which was considered to signify the end of the rest period [37]. Subsequently, the participants were required to continue the sorting process for a further 30 min.

The specific experimental arrangements are as follows:

Experiment A: The participants were required to engage in continuous work for 60 min, in a scenario in which two transport vehicles arrived simultaneously.

Experiment B: The second scenario involved the arrival of two vehicles in succession. After a 30 min sorting period, participants were allowed to rest in a seated position until their physiological signals reached a stable state. They then engaged in another 30 min of work.

The data were collected at five-minute intervals, and the work periods were identified as T1–T12 segments. During the sorting process, parcels were randomly allocated to a desktop location, and participants were only permitted to select the parcel situated in closest proximity to them. Subsequently, the participants were required to traverse a distance of 0.8 m to the designated placement area (designated as area A, B, or C) and correctly position the parcel. To ensure that the work proceeded in an efficient and timely manner, a minimum of 30 parcels had to be sorted within each five-minute interval.

**Figure 1:**
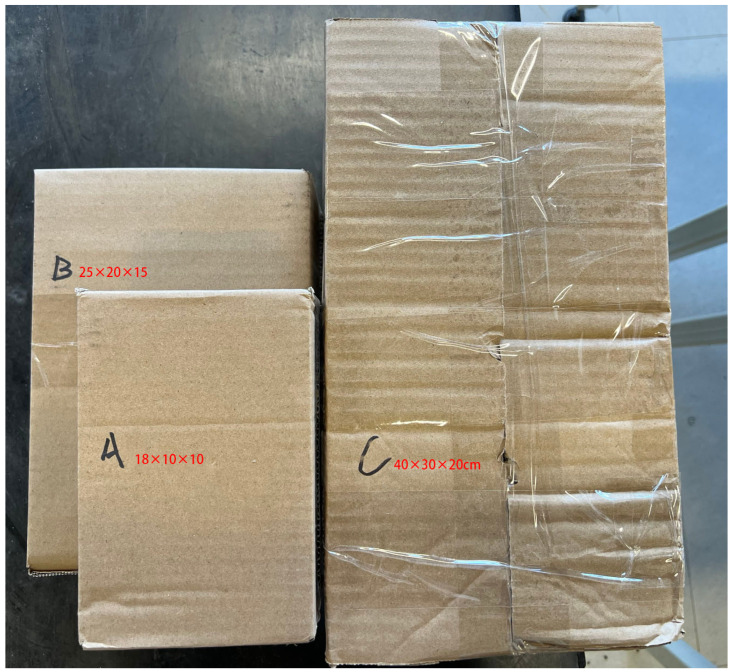
The parcel sorting experiment employed three distinct types of boxes, which were labelled A, B, and C to represent the small, medium, and large sizes, respectively.

**Figure 2:**
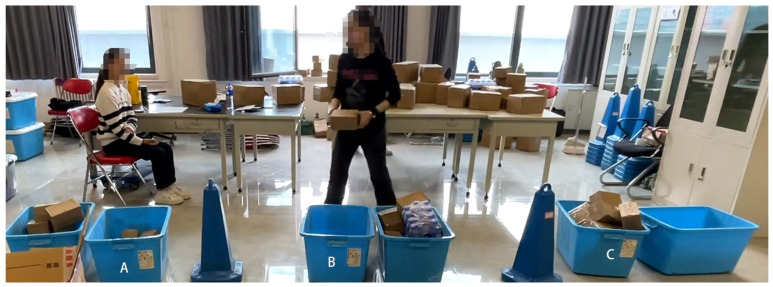
The experimental setup, which shows the environment and tasks. The three blue sorting bins, labelled A, B, and C, are the designated areas for different types of parcels.

The experiment employed cardboard boxes of three common sizes: small boxes (18 × 10 × 10 cm), primarily used for items weighing less than 1 kg, which accounted for 38.54%; medium boxes (25 × 20 × 15 cm), mainly used for items weighing 1 to 3 kg, and representing the highest proportion at 45.32%; and large boxes (40 × 30 × 20 cm), mainly used for items weighing more than 3 kg, accounting for 16.14%. The items that were sorted in the experiment can be observed in Figure 1, while the experimental setting is depicted in Figure 2.

The experiment was conducted on two separate occasions, with a one-month interval between sessions. This time interval was implemented to eliminate any potential influence of experiment A on experiment B. On the day of the experiment, the participants arrived at the laboratory at 10 a.m., having consumed breakfast beforehand. The participants were outfitted with EMG and EDA electrodes to facilitate the collection of data throughout the experiment. The number of items sorted during each work time unit was recorded. After each work time unit, participants were required to complete a subjective fatigue scale, which provided insights into their perceived level of fatigue. Furthermore, participants were questioned about their physiological condition and any localized discomfort they may have experienced. Following the experiment, researchers engaged in interactive discussions with the participants to assess their overall emotional positivity or negativity. During the second intermittent work experiment, the recovery times for EMG and EDA measurements were recorded for each participant. The entirety of the experiment was recorded on video for further analysis and reference.

### 2.3. Assessment Tools

The experiment employed Borg’s Rating of Perceived Exertion (RPE) scale [38] as a subjective fatigue scale to evaluate participants’ perceived fatigue levels. After each sorting period, participants were instructed to complete the scale, offering their assessment of their perceived level of fatigue. The corresponding fatigue levels for each grade are presented in Table 1.

The sympathetic nervous system, which is part of the autonomic nervous system, has a strong influence on the body’s emotional state. Emotional changes can result in increased sweat gland secretion, which in turn affects skin conductivity and causes variations in the skin conductance level (*SCL*) [39]. To supplement and corroborate the physiological data as an indicator, we conducted interactions with participants using the Positive and Negative Affect Schedule (PANAS) [40] after each work unit. This assessment was employed to evaluate the participants’ emotional valence. If there was an increase in negative emotions during the work process, it was considered to be an indication of heightened psychological fatigue.

### 2.4. Physiological Measurements

The experiment utilized the ErgoLAB human–machine environment synchronization platform, developed by Kingfar Technology, as the experimental equipment. The platform facilitated the study of human physiological state changes during activities in various work environments and enabled the collection of data in two modalities simultaneously.

EMG was employed to monitor the electrical activity of the erector spinae muscles. Following the completion of several preliminary experiments, the erector spinae muscle on the right side, which is situated between the L3 and L4 lumbar vertebrae, was selected for monitoring due to its pivotal role in force generation. Red and blue electrode patches were aligned with the muscle fibers and attached to the surface of the skin, while a black electrode was placed at a distance from the test site for reference. Before the formal experiment, we performed the Maximum Voluntary Contraction (MVC) assessment during the vertical spinal muscle test. Subsequently, the software automatically calculated the MVC value by segmenting the time and locating the cursor. For the vertical spinal muscle test, the participant was positioned in a supine position with a slight lumbar curve. Electrodes were placed on the vertical spinal muscle between the right L3 and L4 vertebrae, aligned with the direction of the muscle fibers, while reference electrodes were placed on the left side. Participants were instructed to rest quietly for 5 min to obtain baseline physiological signals in a relaxed state. Participants then engaged in pulling a resistance band with maximal force and maintained this force for 3 s while muscle signals were recorded. This process was repeated three times, with a 2 min rest period between each trial. The recorded maximum force signal was identified as the MVC value for subsequent standardization. The formal experiment began when the electromyographic signals reached a stable state. The device was configured with specific filters, including a high-pass filter with a cutoff frequency of 5 Hz, a band-stop filter at 50 Hz, and a low-pass filter at 500 Hz. Subsequently, data normalization procedures were implemented, followed by rectification using the root mean square method. The sampling rate was set at 1000 Hz and a minimum interval of 1000 ms to ensure the accurate acquisition of the EMG data.

**Figure 3:**
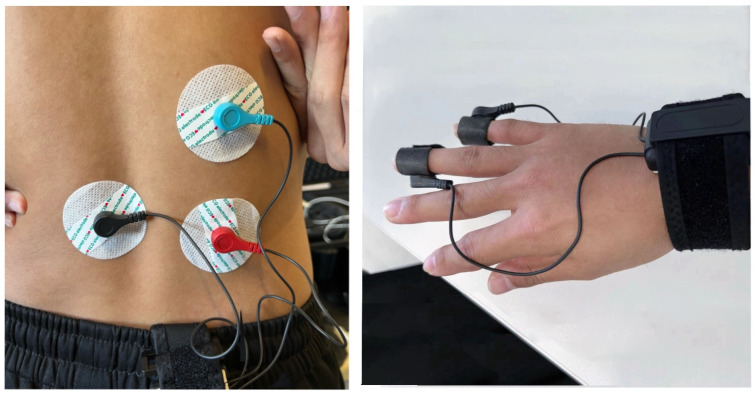
Photo of electrode placement. The photo on the left illustrates the placement of electrodes for the collection of EMG data, while the photo on the right shows the positioning of the EDA data electrodes.

EDA was recorded to reflect the emotional states of the participants by monitoring changes in skin conductivity. To reduce the likelihood of data collection errors resulting from exertion-related compression, electrodes were positioned on the lateral surfaces of the ring and little fingers and secured with medical tape. The electrodes were positioned to minimize data errors, but future research should evaluate how device placement may influence fatigue levels in participants. The root mean square method was employed, and a low-pass filter with a cutoff frequency of 5 Hz was utilized to generate a smooth EDA curve. Figure 3 illustrates the placement of the equipment on the participant.

During the experiment, it was of critical importance to ensure the stability of the physiological measurement signals to determine the length of rest periods. The recovery times required for both the EMG and EDA signals to reach a stable state were recorded. Once both signals indicated stability, it was inferred that the participant’s fatigue had sufficiently subsided, thereby permitting the continuation of subsequent experimental activities.

Throughout the sorting process, the collected raw surface EMG signals manifested as one-dimensional time-voltage signals. To analyze these signals in the frequency domain, the Fast Fourier Transform (FFT) was applied, resulting in the frequency domain that enabled the determination of median frequency (*MF*) values [41,42].

(1) $$MF=\int_{-\infty }^{+\infty }PSD\left(f\right)df=\frac{1}{2}\int_{0}^{f0}PSD\left(f\right)df,$$

where EMG(t) represents the EMG curve, PSD(f) denotes the power spectral density of the surface EMG, and f0 represents the upper-frequency limit. The *MF* represents the middle value of the frequency of electrical signals during muscle contraction. A reduction in *MF* is indicative of the commencement and progression of muscle fatigue. As fatigue develops, the power spectrum typically shifts from higher to lower frequencies, and the firing pattern of muscle fibers changes from fast and intense to slow and less intense. Consequently, a lower *MF* value is associated with a more pronounced physiological fatigue.

The collected EDA signal is a one-dimensional skin conductance signal that does not undergo frequency domain analysis. Also, the mean *SCL* is derived from the tonic data of the EDA signal and is utilized to indicate emotional states [43,44].

(2) $$SCL=\frac{1}{N}\sum_{n=1}^{N}X_{n},$$

where $X_{n}$ is the tonic component of EDA. The *SCL* is a reliable indicator of sympathetic nervous system activity. When individuals are exposed to external stimuli or psychological influences, the sympathetic nervous system is activated, which in turn controls the activity of the sweat glands via postganglionic nerve fibers. This activation increases skin conductance. Variations in *SCL* are indicative of alterations in both emotional and physiological states. Higher *SCL* values indicate greater intensity of sympathetic nervous system activity, which may be indicative of elevated emotional arousal or stress levels.

### 2.5. Data Analysis

The processing of EMG and EDA signals was conducted on the ErgoLAB cloud platform. Descriptive statistics, including means and standard deviations (mean ± SD), were provided. The data’s suitability for analysis was confirmed by the Shapiro–Wilk test, which was used to assess the distribution of the data.

To compare each variable between the two experiments, a repeated measures analysis of variance (RM-ANOVA) was conducted using the Afex package [45,46]. Post hoc tests were performed using the Sidak method implemented with the emmeans package [47]. Additionally, a paired samples *t*-test was conducted using the built-in *t*.test() function in R 4.4.0 to compare mean values for specific conditions. Correlations based on panel data were examined using the methods described by [48]. Statistical significance was set at *p* < 0.05. In addition, slow-motion experimental footage was used to analyze the participants’ action changes.

## 3. Results

### 3.1. Participant Descriptive

The study involved a total of 15 participants, comprising 12 males and 3 females. Detailed demographic information is provided in Table 2 below:

### 3.2. *MF* and *SCL* Difference Analysis

This study focuses primarily on the processing of EMG and EDA signals, which includes filtering and normalization. The filtering process is used to remove noise from the human body or interference caused by movement. The initial values of the subjects under different experimental conditions are shown in Table 3. The processed data of the erector spinae *MF* of the participants are presented in Table 4. The changes in the participants’ *SCL* from T1 to T12 are shown in Table 5.

The results of the RM-ANOVA on the *MF* of Experiment A show a significant effect of time on the *MF* (F(12,108)=13.68, *p* < 0.001, ges=0.404). The generalized effect size (ges) indicates that the time variable accounts for 40.4% of the total variance in *MF*. This finding suggests that time has a significant impact on *MF*. To further investigate specific differences between time points, post hoc pairwise comparisons were performed using the Sidak correction method. These comparisons revealed significant differences in *MF* scores between different time points, particularly between the baseline and several subsequent time points.

Similarly, an RM-ANOVA on the *SCL* for Experiment A indicates that time significantly affects the *SCL* (F(12,108)=43.24, *p* < 0.001, ges=0.617). The results of the Sidak post hoc tests also indicate significant differences between multiple time points. These results confirm that *MF* and *SCL* undergo statistically significant changes during continuous tasks, reflecting participants’ physiological and psychological states of fatigue.

In Experiment B, the analysis of *MF* and *SCL* also shows the significant effects of time on *MF* (F(12,108)=4.84, *p* < 0.001, ges=0.206) and *SCL* (F(12,108)=16.99, *p* < 0.001, ges=0.503). Post hoc tests also indicate significant differences between different time points. Therefore, in Experiment B, *MF* and *SCL* show statistical significance, indicating changes in participants’ fatigue levels.

Table 3 and Table 4 illustrate the changes in *MF* over time in two experimental conditions. In Experiment A, *MF* shows fluctuations and a decrease during T1–T6, followed by a continuous and significant decrease from T7 to T12. Similarly, Experiment B shows an initial decrease in *MF* during T1–T6, a slight recovery in the middle period, but an overall decreasing trend. During T7–T12, *MF* increases initially, but then shows a fluctuating downward trend.

The *SCL* in Experiment A increases significantly from an initial low value, with the upward trend becoming more gradual during the T8–T10 period. In contrast, in Experiment B, the *SCL* shows an upward trend in both the initial and subsequent phases.

To analyze the differences in the mean values of *MF* and *SCL* between the first 30 min and the last 30 min of Experiment B, a paired samples *t*-test was performed. The *MF* (t=−1.30, p=0.23, 95%CI=[−3.5719,0.9645]) showed no significant difference, indicating an effective recovery from physiological fatigue after rest. However, *SCL* (t=2.96, p<0.01, 95%CI=[0.22,1.66]) showed a significant difference between the first 30 min and the last 30 min, with a higher rate of change in *SCL* observed in the first 30 min. In addition, the upward trend in *SCL* gradually leveled off during the T10–T11 period.

Overall, to analyze the differences in the mean values of *MF* and *SCL* between the first 30 min and the last 30 min of the experiment, a repeated measures ANOVA was conducted. During the first 30 min of the experiment, the interaction effect between the experiment and the time period was not significant for *MF*, with significance levels of *F*(2.74, 49.33) = 0.01, *p* = 0.999, ges < 0.001. Similarly, no significance was shown for *SCL*: *F*(2.01, 36.10) = 0.41, *p* = 0.667, ges = 0.009, whereas in the last 30 min of the experiment, the interaction effects between the experiment and the time period became significant for both *MF* and *SCL*. For *MF*, the differences were as follows: *F*(3.19, 57.37) = 5.24, *p* = 0.002, ges = 0.064. For *SCL*, the differences were *F*(2.16, 38.96) = 10.93, *p* < 0.001, ges = 0.134.

### 3.3. Analysis of Rest Periods

**Figure 4:**
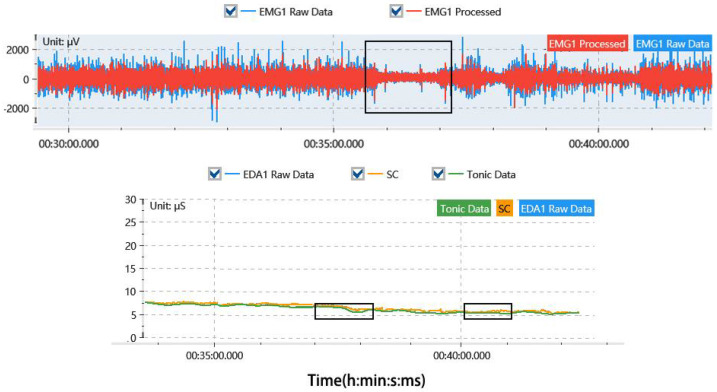
Illustration of physiological signal stabilization, with EMG on the top and EDA on the bottom. EMG1 Raw Data: The original raw EMG data; EMG1 Processed: The filtered and normalized EMG data; EDA1 Raw Data: The original raw EDA data; SC: Skin conductance level; Tonic Data: The tonic component of the EDA.

As shown in Table 6, the time required for EMG and EDA signals to stabilize was recorded for participants in Experiment B after 30 min of continuous work. Notably, there is a significant difference between the recovery times of the EMG and EDA signals. In general, participants exhibited shorter EMG stabilization times compared to EDA stabilization times. The average recovery time was 414.9 s (approximately 6 min and 55 s), with a maximum recovery time of 460 s (7 min and 30 s). These results are depicted in Figure 4, where both EMG and EDA signals show stabilization after the rest period.

### 3.4. Analysis of Changes in Sorting Count

**Figure 5:**
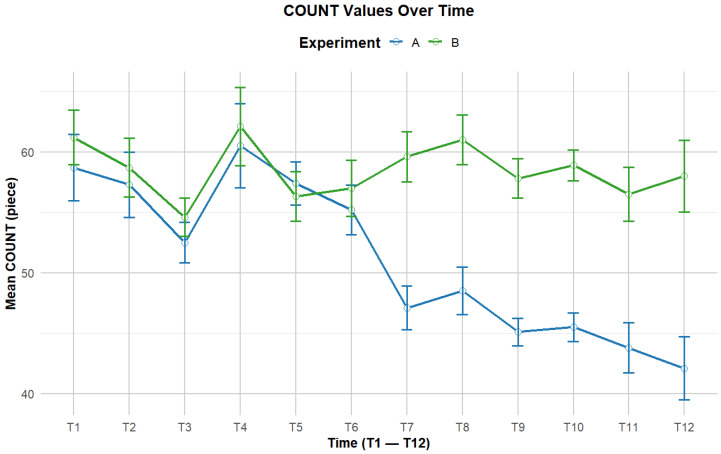
Sorting count over time.

The trend of the average sorting count at different time points for the two experimental modes is shown in Figure 5.

During the period T1–T6, the average sorting count of Experiment A shows a fluctuating trend, first decreasing and then increasing. Similarly, Experiment B maintains this trend during the same period.

As the T7–T12 period, Experiment A’s average sort count continues to decrease, eventually dropping to approximately 42. In contrast, Experiment B’s average sort count remains relatively stable during this period, fluctuating between 56 and 61. This indicates a clear distinction between the two experiments, with Experiment B consistently showing a higher average sort count than Experiment A.

To further analyze the sorting performance before and after the rest period in Experiment B, a paired sample *t*-test was performed on the sorting count in the same time-unit segments, comparing T1 with T7, T2 with T8, and so on. The *p*-values for these comparisons were all greater than 0.05, indicating no significant differences in participants’ performance before and after the rest period.

When we calculated the number of items sorted per minute for both experiments, Experiment A had a total duration of 60 min, resulting in an average sorting efficiency of approximately 10.23 items per minute. For Experiment B, considering the maximum rest period of 460 s, the total duration was 67.5 min, with an average sorting efficiency of 10.40 items per minute. Interestingly, the average sorting efficiency after the rest period in Experiment B was slightly higher that than observed during continuous work.

### 3.5. Panel Data Correlation Analysis

**Figure 6:**
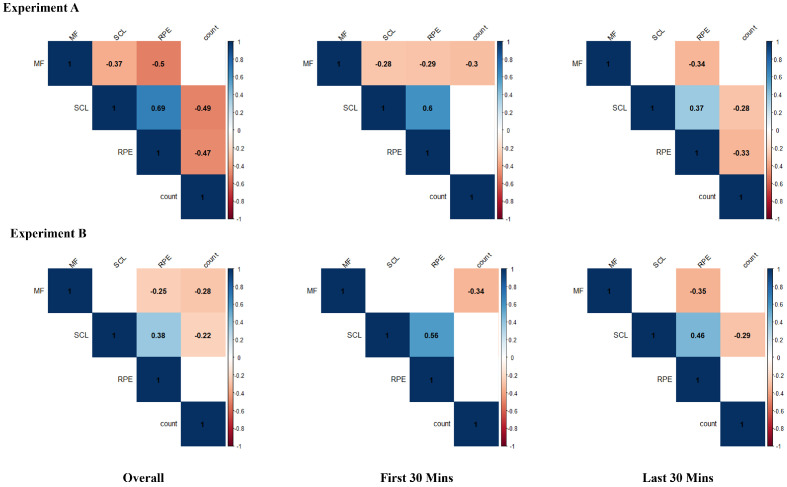
Correlation matrices for different experiments. Only significant correlations are displayed. The numbers in the matrices represent the Pearson correlation coefficients. The figure is organized vertically with Experiment A on top and Experiment B below. The data for each experiment are divided into three segments horizontally, from left to right: total duration, the first 30 min, and the last 30 min.

As shown in Figure 6, a panel data correlation analysis was conducted for both Experiment A and Experiment B, focusing on the total duration, the first 30 min, and the last 30 min. This analysis revealed notable differences in the correlations between variables at different stages of the work.

For Experiment A, the overall analysis revealed a significant negative correlation between the sorting number and both *SCL* and RPE. Specifically, during the first 30 min, *MF* showed a significant negative correlation with all other variables. During the last 30 min, both *SCL* and RPE showed a significant negative correlation with sorting count.

In Experiment B, the overall analysis showed a significant negative correlation between sorting count and both *MF* and *SCL*. During the first 30 min, the sorting count showed a significant negative correlation only with *MF*. During the last 30 min, there was a significant negative correlation between the sorting count and the *SCL*.

### 3.6. Video Analysis

**Figure 7:**
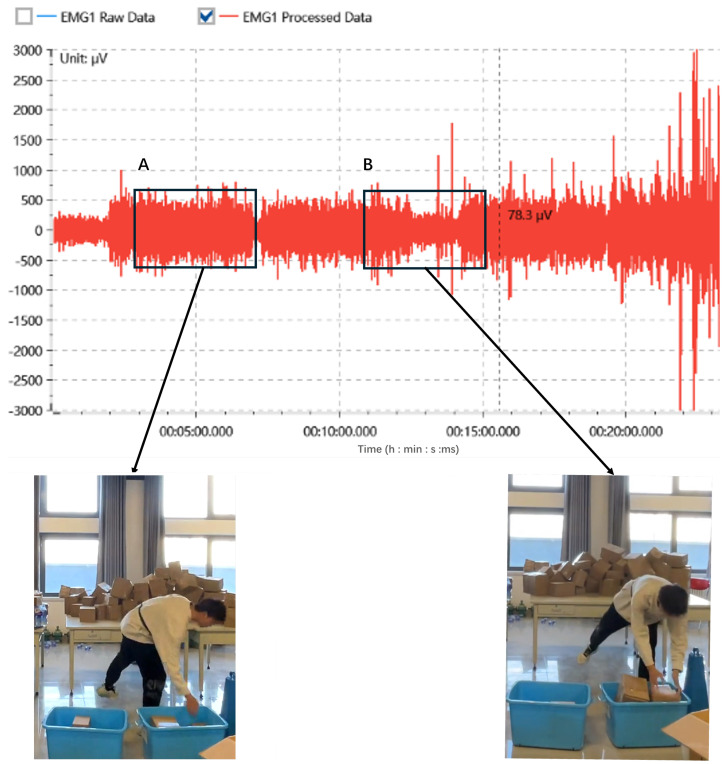
The EMG illustrates the change in movement patterns of the participants during a work task. The corresponding video snapshots from the experiment provide visual evidence of the changes in movement patterns at the highlighted times. A Before change. B After change.

The video analysis indicated significant changes in participants’ actions during time points T3 to T5, as reported by the participants themselves after the experiment, along with observations made by the experimenters during the sessions. A slow-motion review of the footage from this period revealed that participants altered their working methods. Further analysis of the corresponding EMG recordings during this time confirmed these observations, as shown in Figure 7.

## 4. Discussion

The purpose of this study was to replicate the real-world conditions of a parcel sorting center and to examine the performance and fatigue levels of workers during continuous and intermittent sorting tasks. The study involved fifteen participants, all of whom had previous experience in part-time parcel sorting. Two types of tasks were performed: a 60 min continuous sorting task and an intermittent sorting task consisting of two 30 min sorting sessions with a rest period in between. Several physiological measures, including EMG and EDA, as well as data from Borg’s RPE scale and the total count of items sorted per unit time, were collected during the experiment.

### 4.1. Overall Analysis of the Two Experiments

Initial comparisons between the two experiments revealed differences in baseline physiological indicators. These differences could be attributed to variations in participants’ physiological states and their perceptions of the tasks’ difficulty, which can lead to negative emotions such as lethargy. The experiment effectively captured these emotional states, indicating that task complexity and its perception can influence fatigue levels [49].

Overall, the experimental results showed that the trends of *MF* and *SCL* over time were consistent with previous research [50,51,52,53]. These results suggest that prolonged repetitive tasks lead to muscle fatigue, as reflected by a decrease in *MF* and an increase in physiological fatigue. The increase in *SCL* is closely related to increased psychological stress and negative emotions. In this study, participants experienced significant increases in muscle fatigue and negative emotions as work time progressed.

The analysis of Experiments A and B showed that participants in both groups experienced a slight increase in *MF* between T3 and T5, indicating that they experienced some relief from physiological fatigue and an increase in the sorting count during this period. Slow-motion footage of the experiment showed that participants changed their movement patterns during this time, using different muscle groups to complete the tasks. This change was also supported by the EMG recordings. This finding is consistent with previous research on similar repetitive tasks, such as sawing, where workers were found to adjust their muscle use strategies to maintain work efficiency despite muscle instability [54].

### 4.2. Analysis of Experiment A

In Experiment A, the *MF* showed a decrease after T5 to T6 and continued to decrease throughout the experiment, indicating a continuous accumulation of physiological fatigue. Although this downward trend stabilized in the later stages, it still indicated the continued accumulation of muscle fatigue. This finding is consistent with previous research by Merletti and Roy [55], who described the *MF* change curve during prolonged work as an exponential decay curve, where the rate of decline in *MF* slows as fatigue accumulates.

*SCL* increased steadily and stabilized between T10 and T12. Participants reported dizziness, blurred vision, and difficulty concentrating during the latter stages of the task. A study using event-related potentials found that psychological fatigue during or after prolonged cognitive activity impairs workers’ ability to concentrate and focus their attention [31]. Similar symptoms have been observed in vigilance tasks [56], suggesting that as individuals maintain attention and alertness to specific stimuli over time, their ability to detect target stimuli gradually decreases, reaction times increase, and error rates increase—a phenomenon known as vigilance decrement.

### 4.3. Analysis of Experiment B

In Experiment B, participants’ EMG signals stabilized after a short rest period, with *MF* showing no significant difference from the beginning of the experiment. In addition, from T7 to T12, the *MF* of participants in Experiment B were significantly higher than those of participants in Experiment A, indicating that there was a lower degree of physiological fatigue in Experiment B. This suggests that the rest periods built into the experimental design effectively alleviated muscle fatigue. The absence of differences in sorting counts before and after the rest periods suggests that participants’ muscle endurance was restored, reducing the impact of work fatigue on productivity [57].

The *SCL* in Experiment B showed a fluctuating upward trend before and after the rest periods, with a noticeable slowing or decrease in the rate of increase during the latter stages of two work phases. Dawson, Schell, and others suggested that this result was due to the gradual decline in the *SCL* that was typically observed after prolonged repetitive stimulation [58]. The rate of change in the *SCL* was significantly lower after the rest period, and the highest measurements taken after rest were also lower than those taken before rest. This could be attributed to the participants’ increased familiarity with the experimental setup, which may have reduced their emotional distress. Post-experiment interviews revealed that many participants found the tasks easier after the rest period. This is consistent with previous research indicating that increased task proficiency can significantly reduce psychological distress [59].

### 4.4. Subjective Fatigue Analysis

Prior research has demonstrated a strong linear correlation (r = 0.76, *p* < 0.01) between the reduction in mean power frequency (MPF) of surface electromyography signals from the upper trapezius muscle, which is indicative of elevated physiological fatigue, and the increase in RPE. It is important to note, however, that this study focused exclusively on two endurance tasks, each lasting a maximum of six minutes, and did not investigate the potential variations in correlation over prolonged periods of work [60].

In our study, we found that psychological fatigue, as indicated by *SCL*, plays a significant role in shaping subjective fatigue, as measured by RPE. It is noteworthy that during the 30 min post-experiment phase, both *SCL* and *MF* had an impact on RPE. These findings indicate that in the context of short-term, repetitive tasks, RPE may serve as a viable substitute for *SCL*. However, in tasks lasting more than 30 min, both physiological and psychological fatigue have a substantial impact on subjective fatigue. Consequently, relying solely on RPE as a measure for either physiological or psychological fatigue is inadequate. Instead, it is essential to comprehensively consider both factors to accurately comprehend shifts in participants’ RPE throughout the experiment.

### 4.5. Rest Period Study

With regard to the required rest time after 30 min of continuous parcel sorting, the study found that participants needed approximately 415 s (6 min 55 s), with a maximum of 460 s (7 min 30 s), to recover their working capacity and complete their rest period. The recovery rate for physiological fatigue was significantly faster than that for psychological fatigue [58]. Previous studies that only considered physiological fatigue were overly simplistic and failed to fully assess the fatigue states of the participants [61]. In addition, EMG stabilization does not directly indicate recovery of work capacity [62]. By measuring the recovery times of various physiological indicators and checking whether there were differences in sorting efficiency before and after the rest periods, the extent of fatigue recovery was determined [63]. The results confirm that it is reasonable to consider both types of fatigue when setting rest periods. Interestingly, despite the longer rest periods in Experiment B, the average efficiency was still significantly higher than in Experiment A. This suggests that in addition to enhancing workers’ physiological and psychological health, adequate rest can also significantly improve work efficiency, and that setting rest periods based on physiological fatigue recovery can ensure participants remain able to work.

### 4.6. Limitations

It is important to acknowledge certain limitations of this study to ensure a balanced interpretation of the results. While our research provides valuable insights into the relationship between physiological and psychological fatigue and work performance among express terminal sorters, several factors may affect the broader applicability and depth of interpretation.

First, it should be noted that the study primarily involved university students as participants. Although their age distribution is similar to that of express terminal sorters, there may be significant differences in terms of work experience, physiological fitness, and lifestyle habits. For example, long-term express terminal sorters may have developed specific work skills and strategies that affect the accumulation and recovery of fatigue.

The sample size of 15 participants, while consistent with similar studies in the field, may limit our study’s generalizability. For example, studies [16,17,64] used 4 to 12 participants. Although this smaller sample size allowed for detailed monitoring and high-quality data collection, using larger and more diverse samples in future research would strengthen the validity of the findings.

In addition, the duration of the experiment in this study was relatively short, with a maximum of one hour of continuous work. This time may not fully capture the effects of prolonged work on the physiological and psychological fatigue experienced by express terminal sorters. In the express industry, especially during peak periods, workers are often required to work longer hours, which may lead to more severe accumulation of fatigue and potential health problems. Future studies could consider extending the experimental period to a week or more to simulate realistic work cycles and examine the impact of long hours on worker health and efficiency. This would provide a more complete understanding of the relationship between workload, employee well-being, and overall efficiency.

Moreover, a notable limitation of this study is the lack of randomization in the order of experimental conditions A and B. Although we took steps to maintain stability in the experimental environment, such as instructing participants to avoid vigorous physical activity and to ensure they had adequate sleep, the nonrandomized design may still introduce potential biases in the results. The order in which participants experienced the conditions could influence their performance and physiological responses, leading to confounding effects. Future research should consider using a randomized design and rigorously controlling the time intervals between conditions to mitigate these potential biases and increase the generalizability of the results. This approach would help ensure that any observed effects are due to the conditions themselves, rather than the order in which they were presented or the stability of the intervals.

## 5. Conclusions

This study focused on examining changes in the operational efficiency of express terminal sorters under different work arrangements. By analyzing physiological measures, we aimed to understand the mechanisms that affect sorting efficiency at different stages. The results indicate that different fatigue factors influence work efficiency at different stages of the sorting process.

In the 60 min work model, we observed that sorting efficiency during the first 30 min is primarily influenced by physiological fatigue. However, as physiological fatigue intensifies and becomes harder to recover from in the subsequent 30 min, psychological fatigue begins to play a more significant role in affecting sorting efficiency. Therefore, it is important to consider both physiological and psychological factors when studying long-term fatigue.

A significant finding of this study is that the stabilization time of EDA after sorting work may indicate recovery from both physiological and psychological fatigue, thus maintaining the sorting ability of workers. Rest periods of 415–460 s, based on EDA recovery times, effectively support sorting efficiency and participants’ recovery. In addition, we found that RPE reflected participants’ psychological fatigue during short-term work. However, for tasks longer than 30 min, the RPE is influenced by both psychological and physiological fatigue.

By applying physiological measurement techniques to the parcel sorting process, we can identify reference indicators for measuring sorting efficiency and participants’ physiological changes. This has practical implications for setting labor quotas and making informed decisions for express terminal sorters. It provides a scientific basis for establishing appropriate rest periods based on physiological indicators, thereby providing reference points for assessing sorting ability. The findings also underscore the importance of rest in long-duration, high-intensity, repetitive sorting tasks. However, further comprehensive research is needed to validate these findings.

## Figures and Tables

**Table 1:** Borg’s RPE scale for determining the perceived level of fatigue of the parcel sorting workers.

Level of Exertion	PRE Score	Description
No exertion at all	6	No muscle fatigue, breathlessness or difficulty breathing.
Extremely light	7	Very, very light.
	8	
Very light	9	Like walking slowly for a short while. Very easy to talk.
	10	
Light	11	Like a light exercise at your own pace.
Moderate	12	
Somewhat hard	13	Fairly strenuous and breathless. Not so easy to talk.
	14	
Hard	15	Heavy and strenuous. An upper limit for fitness training, as experienced when running or walking quickly.
	16	
Very hard	17	Very strenuous. You are very tired and breathless. It is very difficult to talk.
	19	
Extremely hard	19	The most strenuous effort you have ever experienced.
Maximal exertion	20	Maximal heaviness.

**Table 2:** Demographics of the participants.

Measure	Cohort (*n* = 15)
Age (years)	21.00 ± 1.71
Height (cm)	176.3 ± 5.44
Weight (kg)	65.65 ± 8.13
BMI (kg/m^2^)	21.05 ± 1.75

**Table 3:** Participants’ initial *MF* (Hz) and *SCL* (µS) values.

Participant	1		2		3		4		5	
	*MF*	*SCL*	*MF*	*SCL*	*MF*	*SCL*	*MF*	*SCL*	*MF*	*SCL*
A	23.45	1.98	21.36	1.56	17.53	3.23	17.55	4.56	20.69	1.00
B	20.55	2.01	20.00	2.89	15.05	4.14	16.55	4.64	19.55	1.44
**Participant**	**6**		**7**		**8**		**9**		**10**	
	*MF*	*SCL*	*MF*	*SCL*	*MF*	*SCL*	*MF*	*SCL*	*MF*	*SCL*
A	24.35	0.56	23.85	1.65	25.78	4.78	26	2.32	17.02	1.44
B	23.75	0.78	19.72	2.56	24.68	5.10	25.00	3.23	16.55	2.44
**Participant**	**11**		**12**		**13**		**14**		**15**	
	*MF*	*SCL*	*MF*	*SCL*	*MF*	*SCL*	*MF*	*SCL*	*MF*	*SCL*
A	8.95	3.76	17.35	4.04	14.45	3.87	15.05	3.22	20.25	3.64
B	11.65	4.56	20.25	4.29	13.85	4.10	15.25	2.89	22.35	2.98

A Continuous sorting work. B Intermittent work. *MF* is in Hz, and *SCL* is in µS.

**Table 4:** Erector spinae muscle *MF* (Hz) changes over time.

Time	T1	T2	T3	T4	T5	T6
A	18.07 ± 2.60	15.25 ± 3.05	14.92 ± 4.08	15.11 ± 5.15	16.97 ± 3.69	14.88 ± 3.89
B	16.54 ± 2.79	13.81 ± 2.88	13.40 ± 4.07	13.49 ± 5.08	15.37 ± 3.81	13.48 ± 3.94
**Time**	**T7**	**T8**	**T9**	**T10**	**T11**	**T12**
A	13.11 ± 2.75	13.26 ± 3.03	13.08 ± 3.37	12.63 ± 3.07	12.04 ± 3.12	11.60 ± 3.26
B	15.59 ± 4.19	17.12 ± 4.20	16.75 ± 3.82	16.03 ± 3.30	14.48 ± 3.91	13.94 ± 4.41

*MF* is in Hz.

**Table 5:** *SCL* (µS) changes over time.

Time	T1	T2	T3	T4	T5	T6
A	4.04 ± 1.20	4.92 ± 0.89	5.50 ± 1.13	6.19 ± 0.95	6.64 ± 1.11	7.03 ± 1.18
B	4.29 ± 1.20	5.14 ± 0.73	5.86 ± 0.85	6.53 ± 0.80	7.01 ± 1.37	7.08 ± 1.02
**Time**	**T7**	**T8**	**T9**	**T10**	**T11**	**T12**
A	7.30 ± 1.76	7.79 ± 1.92	8.02 ± 1.97	8.28 ± 1.72	8.31 ± 1.74	8.25 ± 1.71
B	4.11 ± 1.30	4.56 ± 1.27	4.98 ± 1.23	5.39 ± 1.28	5.50 ± 1.33	5.77 ± 1.49

*SCL* is in µS.

**Table 6:** Recovery times (s) for EMG and EDA.

Participant	Resting Periods	
	EMG	EDA
1	158 s	428 s
2	165 s	454 s
3	170 s	449 s
4	140 s	348 s
5	175 s	465 s
6	147 s	385 s
7	165 s	460 s
8	150 s	364 s
9	159 s	432 s
10	145 s	364 s
Mean ± SD	157.4 ± 11.58 s	414.9 ± 45 s

## Data Availability

The data presented in this study are available on request from the corresponding author. The data are not publicly available currently as they are stored on-site at the Beijing Information Science and Technology University, as stipulated in the Ethics application.
